# Fertility restoration of racing mare with persistent corpus luteum

**DOI:** 10.14202/vetworld.2021.2356-2361

**Published:** 2021-09-08

**Authors:** Tjok Gde Oka Pemayun, Imam Mustofa, Laba Mahaputra, Herry Agoes Hermadi, Ngakan Made Rai Wijaya, Sri Mulyati, Suzanita Utama, Tjuk Imam Restiadi, Rimayanti Rimayanti

**Affiliations:** 1 Laboratory of Veterinary Reproduction, Faculty of Veterinary Medicine, Udayana University, Jl. PB Sudirman, Denpasar, Bali 80233 Indonesia; 2 Division of Veterinary Reproduction, Faculty of Veterinary Medicine, Universitas Airlangga, Kampus C Mulyorejo, Surabaya 60115, Indonesia; 3 Head Section of Health and Husbandry of the Indonesian Equestrian Association, East Java Region, Kampus C Mulyorejo, Surabaya 60115, Indonesia; 4 Division of Basic Veterinary Medicine, Faculty of Veterinary Medicine, Universitas Airlangga, Kampus C Mulyorejo, Surabaya 60115, Indonesia.

**Keywords:** estrus, fertility, good health and well-being, ovulation, progesterone, racing mare

## Abstract

**Background and Aim:**

Persistent corpus luteum (PCL) causes anestrus in mares. This study aimed to determine the effect of intrauterine prostaglandin F2α (PGF2α) treatment on PCL of racing mares to restore fertility.

**Materials and Methods:**

Twelve racing mares suspected with PCL were diagnosed using transrectal palpation and confirmed by serum progesterone (P4) concentration measurement. PGF2α was infused intrauterine, followed by serum collection at 24, 48, and 72 h after. Estrous symptoms were monitored, and mating was conducted on day 3 of estrus with an earlier injection of 8.4 μg gonadotropin-releasing hormone twice a day. Transrectal palpation was performed on days 21-30 to observe the corpus luteum. Pregnancy diagnosis was performed rectally on 40-45 days post-mating and confirmed using Doppler ultrasound scanning.

**Results:**

Eleven of the 12 mares had PCL. There was a dramatic reduction in the P4 concentration following PGF2α treatment of mares with PCL. All mares exhibited estrus 2.6±0.55 days post-treatment with a P4 concentration of 0.12±0.12 ng/mL. Rectal palpation and P4 concentration on 21-30 days after estrous onset showed that all mares were ovulating. The evaluation of P4 concentration on days 40-45 post-mating showed that all mares were still in the luteal phase. However, the pregnancy rate was only 54.5% based on rectal palpation and Doppler ultrasound scanning.

**Conclusion:**

Treatment of PCL in racing mares with an intrauterine infusion of PGF2α restored the estrous cycle and induced ovulation and pregnancy.

## Introduction

Horses are seasonally polyestrous, and the fertility traits of mares are affected by environmental factors [1]. The estrous cycle of mares differs between 18 and 22 days, with an average of 21 days [2]. The corpus luteum (CL) is relapsed by prostaglandin F2α (PGF2α) secreted by endometrial around days 13-16 post-estrus in non-pregnant mares. The mares in good health and well-being are expected to reach a reproductive efficiency of one foal per year if there is no postpartum pathological problem. However, much happens during postpartum anestrus and anestrus in mares caused by persistent CL (PCL) [3]. This is because of the extended lifespan of unovulated follicles caused by the absence of the luteinizing hormone surge. Therefore, cumulus cells are directly converted to lutein cells, and they produce progesterone (P4) without ovulation [4]. Delayed ovulation results in immature CL, which in turn results in early embryonic death, and disturbance of the endometrium that diminishes or fails to release PGF2α. Hence PCL can be treated by the administration of PGF2α [5].

To induce luteolysis, a single dose of PGF2α intramuscular treatment is routinely used permitting a subsequent return to the estrus of mares [6]. However, by intramuscular administration, the migration of PGF2α from the injection site to the ovaries of a mare is through the whole body (systemically) [7], which requires a high dose. There has not been reported use of intrauterine PGF2α for estrus induction to restore fertility in racing mares with PCL.

Therefore, this study aimed to determine the effect of intrauterine PGF2α treatment in racing mares with PCL to restore fertility.

## Materials and Methods

### Ethical approval

The experimental procedure was approved by No. 266/HRECC.FODM/VI/2019, by the Animal Care and Use Committee, Universitas Airlangga, Surabaya, Indonesia.

### Study period and location

This study was conducted from July to November 2020 at Trawas stable, Mojokerto, and Kenjeran stable, Surabaya, East Java. Trawas stable is located at latitude S 7° 39’ 59.3568”, longitude E 112° 35’ 15.8316”, and altitude +837 meters above sea level (ASL). The climate of Trawas is wet tropical with significant rainfall most months and a short dry season. The relative humidity is 63.91-90.15 %, and temperature is ±20.6 °C, yearly rainfall is about ±3089 mm, and ±210 rainy days per year. Kenjeran Stables is located at latitude S 7° 14’ 57.01”, longitude E 112° 45’ 2.99”, and altitude one meter ASL. Surabaya is a dry tropical climate with humidity 68%-84%, average temperature ±28°C, and yearly rainfall is about ±848 mm, and ±174 rainy days per year. Meanwhile, serum progesterone measurement was conducted at Sub Laboratory of Endocrinology, Laboratory of Veterinary Obstetrics, Division of Veterinary Reproduction, Faculty of Veterinary Medicine, Universitas Airlangga.

### Animals

Twelve mares (after 2-5 years as racing horses) were used for this study. The mares were bred in Trawas stable, Mojokerto and Kenjeran Stables, Surabaya, East Java, Indonesia. These mares were the patients of the Health and Husbandry Section of Horse Race Club, East Java Region.

### PCL diagnosis

The mares were diagnosed with PCL based on behavioral observation and history of non-cycling and non-return to estrus 2-4 months post-mating. The rectal palpation was performed for the presence of CL and confirmed by the measurement of serum P4 concentration before treatment [8].

### Serum collection and P4 concentration measurement

Mares’ blood samples (5 mL) were obtained from the jugular vein before treatment, at 24, 48, and 72 h post-treatment; 21-30 days post-estrus; and 40-45 days post-mating. P4 was measured using an enzyme-linked immunosorbent assay (ELISA Kit, GBC, Taiwan).

### Treatment, estrus detection, mating, and pregnancy diagnosis

The PCL mares were treated using 5 mg PGF2α (Lutalyse, Upjohn) dissolved in 3 mL aqua dest and then infused into the uterine lumen using a 24G Foley catheter. Estrus detection was based on visual observation and intravaginal examination. The mare in standing heat exhibited vulvar winking followed by urination, a clinical manifestation which was absent in other mammals. The intravaginal examination was performed using a vaginoscopy (Polansky, MediTools Pty Ltd, Australia) two days after treatment to detect cervical dilation and the presence of cervical discharge. The mare did not exhibit cervical dilatation nor cervical discharge on day 2; estrus detection was repeated on day 3. The estrous mares were confirmed the day after with a teaser stallion [9,10].

Natural mating was conducted on day 3 of standing heat by previously providing an intramuscular injection of 8.4 μg synthetic gonadotropin-releasing hormone (GnRH) (Buserelin acetate, Concepts, AgroVet market, Peru) twice a day. Transrectal palpation was conducted on days 21-30 after mating to palpate the presence of the CL on the ovaries and to determine the position of CL in the left or right ovary. Pregnancy diagnosis through transrectal palpation was conducted on 40-45 days post-mating and confirmed using a Doppler ultrasound [9,11] BT-200 scanner (Bistos, South Korea).

### Statistical analysis

Statistical analyses were conducted at a 95% significance level using Statistical Package for the Social Sciences version 23.0 (IBM Corp., Armonk, NY, USA). Data of progesterone concentrations on days 0, 4, 48, and 72 were studied using a general linear model repeated measures [12]. Meanwhile, analysis of data frequency of estrus and pregnancy of mare were studied using Chi-square test [13].

## Results

Transrectal palpation diagnosis of PCL suspected mares based on clinical manifestations showed that all mares had CL in their ovaries. Furthermore, examination showed that 91.7% (11/12) of the mares were PCL positive with a range of serum P4 concentrations of 2.6-9.5 ng/mL (average 5.03±2.68 ng/mL) on day 0 (before treatment). Meanwhile, 8.3% (1/12) was negative for PCL with a P4 concentration of 0.5 ng/mL.

A quarter decrease in serum P4 concentration was noticed from pre-treatment to 24 h post-treatment (p<0.01), 1/8 reduction from pre-treatment to 48 h post-treatment (p<0.01), and a reduction by half from 24 to 48 h post-treatment (p<0.05). Meanwhile, there was no significant (>0.05) decrease in serum P4 concentration 48-72 h after PGF2α treatment (Table-1).

**Table 1:** Serum P4 concentrations (ng/mL) before and after PGF2α treatments.

Parameter	Before (Day 0)	Hours after PGF2α treatment		

		24	48	72
Average	5.03±2.68^a^	1.33±1.01^b^	0.61±0.65^c^	0.13±0.11^c^
Range	2.6-9.5	0.1-2.7	0.00-1.7	0.00-0.4
Decreased concentration (%)	-	73.56	87.87	97.42

Different superscripts in the same row were significantly different (p<0.05) in general linear model repeated measure test. PGF2α=Prostaglandin F2α

All PCL diagnosed mares exhibited estrous signs after PGF2α treatment with serum P4 concentrations <0.2 ng/mL. The estrous rate was 36.4% (4/11) at 48 h and 63.6% (7/11) at 72 h after PGF2α treatment, with 2.6±0.5 days average (Table-2). All mares were ovulating based on the finding of CL in their ovaries in the transrectal examination on 21-30 days after estrous onset, P4 concentration was higher than 1 ng/mL. Those CLs’ distribution was 72.7% (8/11) in the right ovary and 27.3% (3/11) in the left ovary. The average P4 concentrations of mares on 40-45 days post-mating were 2.80±0.59 ng/mL in the range of 1.9-3.6 ng/mL. The P4 concentration of mares based on the position of CL was 2.82±0.65 ng/mL in the right ovary and 2.7 ng/mL in the left (Table-3). The pregnancy rate was 54.5% (6/11), given at the right uterine horn was 45.5% (5/11), and at the left uterine horn was 9% (1/11). Doppler ultrasound scanning showed two digital wave groups: 59-73 beats per minute (bpm) and 181-210 bpm.

**Table 2:** Estrous responses and P4 concentration at estrus after PGF2α treatment.

Parameter	Hours after PGF2α treatment		

	24	48	72
Estrous mares	0	36.4% (4/11)^b^	63.6% (7/11)^a^
P4 concentration at estrus (ng/mL)	-	0.075±0.096	0.16±0.14

Different superscripts in the same row were significantly different (p<0.05) in the Chi-square test. PGF2α=Prostaglandin F2α

**Table 3:** The fertility of mares after PGF2α treatment.

Parameter	Position		Total

	Right ovaries/horns	Left ovaries/horns	
Developing CL (%)	72.7% (8/11)^a^	27.3% (3/11)^b^	100% (11/11)
P4 averages (ng/mL)	2.82±0.65	2.7	2.80±0.59
Pregnancy rate	45.5% (5/11)^a^	9.0% (1/11)^b^	54.5% (6/11)

Different superscripts in the same row were significantly different (p<0.05) in the Chi-square test. PGF2α=Prostaglandin F2α

## Discussion

The CL is a pivotal structure for controlling the estrous cycle and maintaining pregnancy [14]. The CL is formed after ovulation and usually works for 14-15 days in non-pregnant mares. It is then regressed because of the prostaglandin release from the endometrium. A sudden and complete reduction in P4 concentrations in cycling mare due to luteolysis began on day 13, whereas in the PCL mares, the P4 concentrations slowly declined after day 12 and not fully decreased to the basal concentrations [5]. The corpora lutea that failed to regress at the normal time were considered pathologically persistent CL. The CL persisted for 2-3 months, and the mare did not show estrous behavior [7]. Of the 12 mares diagnosed with PCL, one was not in the PCL category because of a P4 concentration of 0.5 ng/mL (day 0).

Meanwhile, the other 11 mares were confirmed to have PCL with a range of serum P4, concentrations of 2.6-9.5 ng/mL (Table-1). PCL diagnosis was conducted using behavioral observations, ovarian transrectal palpation, and measurement of P4 concentrations [8]. Mares with PCL have a good tone of the cervix and uterus on transrectal palpation [15], and P4 concentrations of >1.0 ng/mL, indicating luteal activity [16].

### P4 concentrations before and after PGF2α treatment

PGF2α is widely used in practice to control heat cycles in the mare. PGF2α, available as natural or synthetic, has a unique function of destroying the CL [5]. Sudden decreases of P4 concentration follow the regression of CL. The P4 basal concentration causes the loss of negative feedback to the hypothalamus, the release of GnRH followed by the release of follicle-stimulating hormone (FSH) from the anterior pituitary. The FSH induces follicle development, but when the estrogen derived from the follicles is quantitatively enough in the bloodstream, clinical symptoms of estrus are shown [2].

Hormone concentrations are highly vital to the reproductive efficiency of mares [17]. Here, serum P4 concentrations were reduced from day 0 to 24 and 48 h following PGF2α treatment and stabilized in 48-72 h. The reduction in the P4 percentage was getting more significant daily (Table-1). The average P4 concentration on day 0 was 5.03±2.68 ng/mL, which means that the mares were positive for PCL. Previously, a report showed that at the beginning of luteolysis, the P4 concentration in PCLs was 5.0±0.5 ng/mL [4].

In this study, the mare’s PCL treatment was performed using intrauterine infusion of PGF2α. The PGF2α travels to the ovary and CL as the target. The luteal cell membranes are highly efficient in capturing the PGF2α molecules [5]. Luteolysis caused the loss of lutein cells steroidogenic capacity by apoptotic or non-apoptotic mechanisms due to disintegration of the CL [2]; thereby P4 concentration reduced fast and was subsequently followed by folliculogenesis and estrus [18].

### Estrous responses after PGF2α treatment

Mares with PCL possessed a defect in PGF2α secretion [7]. Therefore, the administration of PGF2α could be used to prompt luteolysis and allow a subsequent return to estrus [6]. Here, all mares showing estrous signs, including excitement, sweating, and mild colic symptoms following PGF2α treatment, were confirmed by blood serum P4 concentrations of <0.2 ng/mL (Table-2). Moreover, an earlier study [19] reported that following 20-35 min PGF2α administration, the mares exhibited restless, sweating, spasm of pelvic muscle, and mild colic symptoms. The percentage of mares that showed estrous signs on day 2 was 36.36% (4/11), and those on day 3 were 63.63% (7/11), on an average of 2.6±0.5 days post-PGF2α treatment (Table-2). The onset of mare estrus after PGF2α was 2.5 days [20], and ovulation occurred 8.4 days following PGF2α treatment [6].

### Fertility of mares after PGF2α treatment

Indicator of mare fertility, among others, is the development of CL following estrus and ovulation. Once ovulation occurs, granulosa and theca cells undergo functional and structural changes; they form CL and produce P4 [21,22]. All of the mares with PCL treated with PGF2α followed by buserelin (synthetic GnRH) injection on day 2 of estrous signs in this study revealed ovulation, indicated by the finding of CL on transrectal palpation, and confirmed by P4 concentration (Table-3). Single injection of buserelin in delayed estrus of a mare effectively induces ovulation [10].

The distribution of CL formation was 72.7% (8/11) in the right ovary and 27.3% (3/11) in the left ovary. This is because the mare’s right ovary is more active than the left ovary. Follicle density, pre-antral follicles per ovarian fragment, and area of ovary structure (antral follicles and CL) of the right ovary of the mare were higher than that of the left ovary [23]. This result differed from the previous study [24], which reported that the relationship between follicle development and regressed CL was 8% ipsilateral, and 24% was contralateral [7]. These differences can be resulted from the small number of data in this study.

All mares treated in this study showed a <0.5 ng/mL P4 concentration during the estrus (Table-2) and 2.80±0.59 ng/mL with a range of 1.9-3.6 ng/mL on 40-45 days post-mating (Table-3). The low concentration of P4 at estrus followed by a higher P4 concentration starting 4 days after the onset of estrus indicated ovulation followed by CL development [25]. The CL was detected rectally in the ovaries of mares on 21-30 days post-estrus.

Although all treated mares with PCL ovulated, unfortunately, the pregnancy rate was 54.5% (6/11) only. The pregnancy rate in this study was lower than those in Arabian mares (74%) [9], although the breeding in this study was performed using natural mating. The sperm concentration, total number of sperm, and percentage of progressively motile sperm in the ejaculate were higher in natural mating than in artificial insemination [26]. A low-quality ovum obtained from the restoration of the ovarian function following PCL may cause the pregnancy failure. A study reported that P4 concentration at the beginning of luteolysis in mares with PCL was lower than those in cycling mares [4], resulting in embryos with a lower quality grade, smaller diameter, and earlier embryo stage compared to normal embryos [27].

Embryo development can be hampered following improper progestin supply when the embryo reaches the uterus [21]. Adequate P4 concentrations are vital mediators of the appropriate embryo-maternal environment during early pre-implantation embryo development [28,29]. The 45.45% of mares may fail to become pregnant in this study because of inadequate P4 concentration during post-mating. All those mares have a P4 concentration of 1.9-3.6 ng/mL on 40-45 days. The endogenous P4 concentrations of >4.0 ng/mL are considered adequate to maintain pregnancy. Mares with P4 concentrations <4.0 ng/mL were at risk of pregnancy loss [20]. The high P4 concentrations were vital for inhibiting myometrium contraction [30] because the connection between the trophoblast layer of the allantochorion and the endometrium’s luminal epithelium was less stable before day 40 of pregnancy [31].

Here, mares’ pregnancy diagnosis was performed by transrectal palpation and confirmed using Doppler ultrasound scanning. Transrectal palpation accurately determined 40-45 days of mare pregnancy, based on the finding of a round-shaped amnion sac (6-10 cm in diameter) [32]. On Doppler ultrasound scanning, there were two groups of digital waves. The digital wave of 59-73 bpm was the uterine artery pulse and 181-210 bpm was the fetal heartbeat. The 1^st^ time fetal heartbeat could be monitored on day 24 of the pregnancy period [33] and can reach >150 bpm at the subsequent pregnancy periods [34]. Meanwhile, the lower digital wave was defined as a uterine artery pulse, slightly different between early and later pregnancy but not found in non-pregnant mares [35].

There are few pharmacological methods to improve the function of the CL to sustain the pregnancy of the mares [14]. The progestin injection [21] or human chorionic gonadotropin promised increased pregnancy rates and reduced pregnancy loss [15]. The demerits of this study are that it did not administer the addition of hormones to maintain the pregnancy. The injection of GnRH that was conducted before mating improved the ovulation rate. Its effects were only for a short time and theoretically would not improve CL function. Post-mating treatment to ensure P4 concentration adequacy in circulation should concern future PCL treatment of mares.

## Conclusion

The intrauterine PGF2α treatment on racing mares with PCL restored estrous cycle, ovulation, and pregnancy after mating. However, the pregnancy rate was low (54.5%).

## Authors’ Contributions

TGOP, LM, and IM: Conception and design of the study. TGOP, LM, HAH, NMRW, and TIR: Acquisition of data. IM, SM, SU, and RR: Analysis and interpretation of data. TGOP, LM, and RR: Drafted the manuscript. IM, SU, and TIR: Critical review/revision. SU and SM: ELISA. All authors read and approved the final manuscript.
